# Ex vivo identification and characterization of a population of CD13^high^ CD105^+^ CD45^−^ mesenchymal stem cells in human bone marrow

**DOI:** 10.1186/s13287-015-0152-8

**Published:** 2015-09-07

**Authors:** Carmen Muñiz, Cristina Teodosio, Andrea Mayado, Ana Teresa Amaral, Sergio Matarraz, Paloma Bárcena, Maria Luz Sanchez, Iván Alvarez-Twose, María Diez-Campelo, Andrés C. García-Montero, Juan F. Blanco, Maria Consuelo Del Cañizo, Javier del Pino Montes, Alberto Orfao

**Affiliations:** Department of Medicine and Cytometry Service (NUCLEUS), Cancer Research Center (IBMCC, USAL-CSIC), Institute for Biomedical Research of Salamanca (IBSAL), University of Salamanca (USAL), Salamanca, Spain; Spanish Net on Aging and Frailty (RETICEF) Instituto de Salud Carlos III, Madrid, Spain; Department of Immunology, Erasmus MC, University Medical Center Rotterdam, Rotterdam, The Netherlands; The Molecular Pathology group, Institute of Biomedicine of Seville - Hospital Virgen del Rocio, Seville, Spain; Centro de Estudios de Mastocitosis de Castilla La Mancha, Hospital Virgen del Valle, Toledo, Spain; Hematology Service, Hospital Universitario de Salamanca and IBSAL, Salamanca, Spain; Orthopedics Service, Hospital Universitario de Salamanca and IBSAL, Salamanca, Spain; Rheumatology Service, Hospital Universitario de Salamanca and IBSAL, Salamanca, Spain; Centro de Investigación del Cáncer, Campus Miguel de Unamuno, 37007 Salamanca, Spain

## Abstract

**Introduction:**

Mesenchymal stem cells (MSCs) are multipotent cells capable of self-renewal and multilineage differentiation. Their multipotential capacity and immunomodulatory properties have led to an increasing interest in their biological properties and therapeutic applications. Currently, the definition of MSCs relies on a combination of phenotypic, morphological and functional characteristics which are typically evaluated upon in vitro expansion, a process that may ultimately lead to modulation of the immunophenotypic, functional and/or genetic features of these cells. Therefore, at present there is great interest in providing markers and phenotypes for direct in vivo and ex vivo identification and isolation of MSCs.

**Methods:**

Multiparameter flow cytometry immunophenotypic studies were performed on 65 bone marrow (BM) samples for characterization of CD13^high^ CD105^+^ CD45^–^ cells. Isolation and expansion of these cells was performed in a subset of samples in parallel to the expansion of MSCs from mononuclear cells following currently established procedures. The protein expression profile of these cells was further assessed on (paired) primary and in vitro expanded BM MSCs, and their adipogenic, chondrogenic and osteogenic differentiation potential was also determined.

**Results:**

Our results show that the CD13^high^ CD105^+^ CD45^−^ immunophenotype defines a minor subset of cells that are systematically present ex vivo in normal/reactive BM (n = 65) and that display immunophenotypic features, plastic adherence ability, and osteogenic, adipogenic and chondrogenic differentiation capacities fully compatible with those of MSCs. In addition, we also show that in vitro expansion of these cells modulates their immunophenotypic characteristics, including changes in the expression of markers currently used for the definition of MSCs, such as CD105, CD146 and HLA-DR.

**Conclusions:**

BM MSCs can be identified ex vivo in normal/reactive BM, based on a robust CD13^high^ CD105^+^ and CD45^−^ immunophenotypic profile. Furthermore, in vitro expansion of these cells is associated with significant changes in the immunophenotypic profile of MSCs.

**Electronic supplementary material:**

The online version of this article (doi:10.1186/s13287-015-0152-8) contains supplementary material, which is available to authorized users.

## Introduction

Mesenchymal stem cells (MSCs) are nonhematopoietic multipotent stem cells that have the ability for self-renewal and multilineage differentiation [1]. These cells were first described as residing in the bone marrow (BM), which is currently the most extensively studied source for MSCs; however, MSCs have also been successfully isolated from tissues other than BM (e.g., umbilical cord blood [2], the placenta [3], amniotic fluid [4], adipose tissue [5], lung [6], skeletal muscle [7] and the dental pulp [8]). Due to their multipotential capacity [1] and immunomodulatory properties [9, 10], an increasing interest has emerged about the biological properties and the potential clinical application of these cells, as they may represent a potential source for cell-based therapy for tissue repair [11, 12] and for suppressing autoimmunity [13]. In addition, MSCs may also play an important role in the pathogenesis of several diseases, including hematological disorders such as multiple myeloma [14], chronic myeloid leukemia [15] and myelodysplastic syndromes [16, 17].

At present, the identification and definition of MSCs are both based on a combination of phenotypic, morphological and functional characteristics, summarized into three minimal criteria by the International Society for Cellular Therapy (ISCT) [18]. Based on these criteria, MSCs must lack expression of hematopoietic markers (CD19 or cyCD79a, CD11b or CD14, CD45, CD34 and HLA-DR), and show positivity for several other proteins (CD73, CD90 and CD105). Furthermore, MSCs must also be capable of adhering to a plastic surface when maintained in standard culture conditions, and to differentiate to at least three different cell lineages (i.e., osteoblastic, adipocytic and chondrocytic lineages) [18].

Despite the above criteria have contributed to the standardization of MSC studies, the need for in vitro expansion of these cells prior to their characterization has been suggested to potentially modify their immunophenotypic, functional or even genetic features during culture [17, 19–22]; such changes could contribute to explain, at least in part, discrepant results observed in the literature about the characteristics of MSCs [17, 22–27]. Therefore, at present there is a great interest in providing markers for direct in vivo and ex vivo identification and isolation of MSCs [28–31]. In this regard, several studies have identified markers that could be used for positive selection of BM MSCs, such as the nerve growth factor receptor (CD271), the mesenchymal stem cell antigen 1 (MSCA-1), STRO-1, SSEA-4 and the CD13 ectoenzyme [32–34], in addition to CD73, CD90 and CD105. However many of these markers do not provide a clear-cut distinction between MSCs and other BM cells; due to their heterogeneous expression, their overlap with other BM cell populations, or the fact that they have not been systematically used for the identification of MSCs.

In the present study we identified (ex vivo) the CD13^high^ CD105^+^ CD45^–^ immunophenotype to define a minor population of BM cells that show immunophenotypic, morphologic and functional features which are fully compatible with those of MSCs. Further comparison of the ex vivo immunophenotypic features of such BM MSCs versus those of in vitro expanded MSCs, obtained either through standard isolation procedures or by flow cytometry-based sorting of CD13^high^ CD105^+^ CD45^–^ cells, showed that the immunophenotypic profile of MSCs undergoes significant changes during in vitro expansion, such changes including downregulation of CD10, CD13 and HLA-DR and upregulation of CD105 and CD146.

## Materials and methods

### BM samples

A total of 65 freshly obtained ethylenediaminetetraacetic acid (EDTA)- or heparin-anticoagulated normal (n = 9) and reactive (n = 56) BM aspirates were obtained from an identical number of individuals (30 males and 35 females; median age 51 years, range 22–76 years) at the University Hospital of Salamanca, Spain, and the Mast Cell Unit of the Hospital Virgen del Valle, Toledo, Spain. Normal BM samples were obtained from healthy donors, while reactive samples corresponded to patients undergoing BM aspiration due to suspected mastocytosis or other hematological disorders, but who did not exhibit any clonal hematological disease. The study was approved by the local Ethics Committee, Comisión de Bioética del Centro de Investigación del Cáncer—IBMCC (USAL-CSIC). In all cases, informed consent was obtained prior to the study, according to the guidelines of the local Ethics Committee, and all experiments were performed following the Declaration of Helsinki.

### Ex vivo multiparameter flow cytometry immunophenotypic studies

**Fig. 1:**
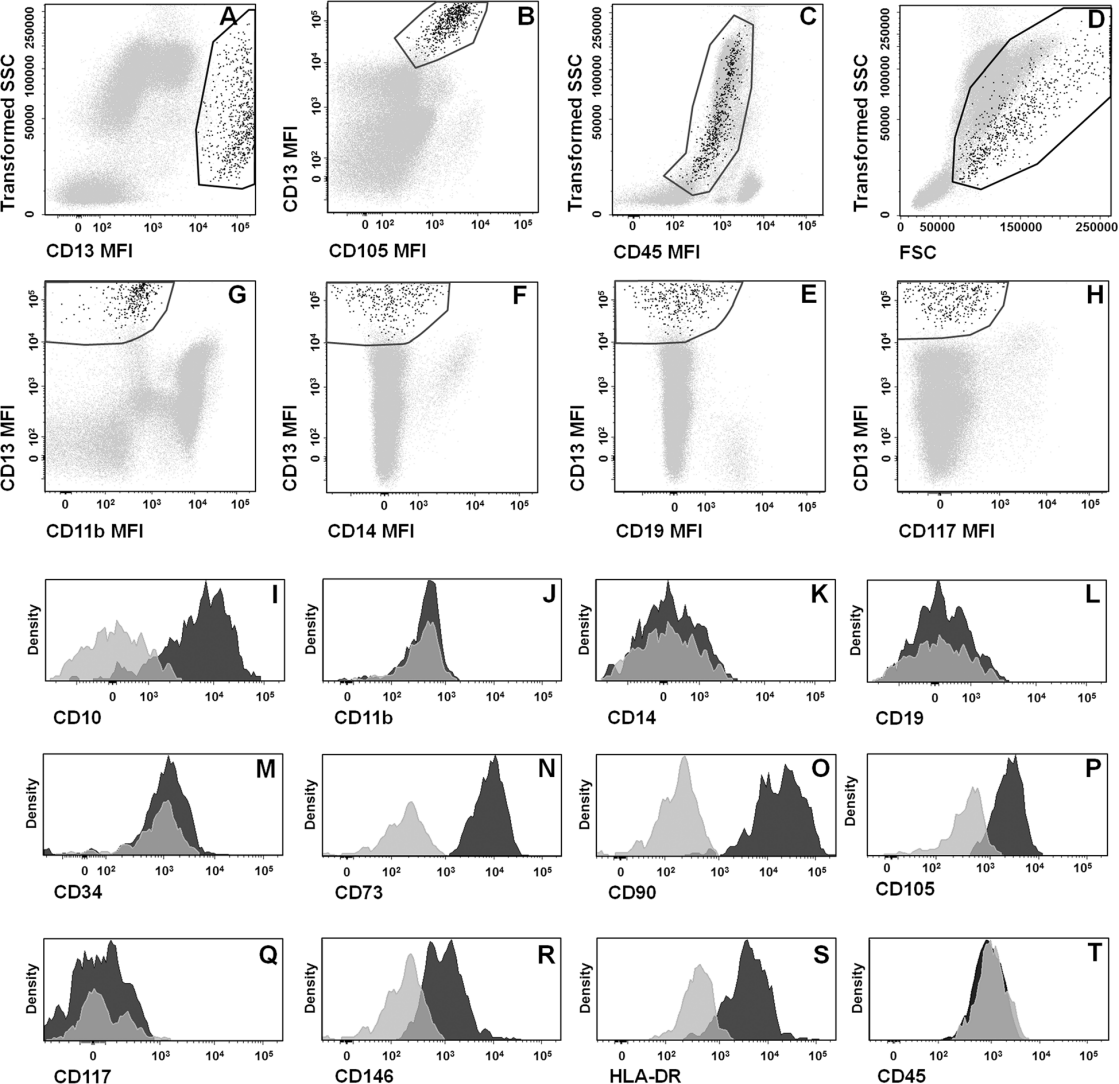
Representative bivariate dot plots and single parameter histograms illustrating the gating strategy and the ex vivo immunophenotypic characteristics of bone marrow CD13^high^ CD105^+^ CD45^–^ cells. **a**–**h** Gating strategy used for the identification of these bone marrow cells based on their unique CD13^high^ CD105^+^ CD45^–^ and high but heterogeneous light scatter features. **i**–**t** Immunophenotypic features of bone marrow CD13^high^ CD105^+^ CD45^–^ cells from a representative healthy donor. Baseline autofluorescence and expression levels for each protein are indicated in the histogram plot in *gray* and *black*, respectively, using the overlay histogram function of the Infinicyt software. *SSC* sideward light scatter, *MFI* median fluorescence intensity (arbitrary units scaled from 0 to 262,000)

Multiparameter flow cytometry (MFC) immunophenotypic studies were performed on BM aspirated samples, processed under sterile conditions within the first 24 hours after they were collected. For ex vivo immunophenotypic characterization of BM MSCs, a direct immunofluorescence stain-and-then-lyse technique was used, as previously described in detail [35]. Eight color combinations of monoclonal antibodies (MoAb) were used to identify and characterize BM MSCs (Additional file 1: Table S1). For each sample, a minimum of 1.5 × 10^6^ cells were analyzed per antibody combination using a FACSCanto II flow cytometer (Becton Dickinson Biosciences, San Jose, CA, USA) equipped with the FACSDiva software (Becton Dickinson). Identification of MSCs was performed based on CD13^high^ and CD105^+^ expression in the absence of CD45 and high but heterogeneous light scatter features (Fig. 1). For data analysis, the INFINICYT software (Cytognos SL, Salamanca, Spain) was used. Expression of individual markers was recorded both as percentage of positive cells and as median fluorescence intensity (MFI; arbitrary fluorescence units scaled from 0 to 262,000) after subtracting the baseline autofluorescence levels observed for MSCs in the corresponding fluorescence detector.

### MSC isolation, expansion and culture

Isolation and expansion of MSCs was performed on 14 heparin-anticoagulated BM samples. Prior to mononuclear cell (MNC) isolation, autologous BM plasma was obtained by centrifugation for (10 minutes at 1200 g). Low density BM MNC were then isolated using a Biocoll density gradient centrifugation step (Biochrom AG, Berlin, Germany). Afterwards, MNC were washed and resuspended in phosphate-buffered saline (PBS) supplemented with 10 % fetal bovine serum (FBS; Gibco, Life Technologies, Paisley, UK). To isolate CD13^high^ CD105^+^ CD45^–^ cells (i.e., MSC fraction), a four-way fluorescence-activated cell sorter (FACSAria, Becton Dickinson) equipped with the FACSDiva software was used. Before sorting, cells were stained with the CD45 pacific orange (PacO)/CD105 fluorescein isothiocyanate (FITC)/CD73 phycoerythrin (PE)/CD34 peridinin chlorophyll protein (PerCP)–cyanin 5.5 (Cy5.5)/CD117 PE–cyanin 7 (Cy7)/CD13 allophycocyanin (APC) combination of MoAbs for 30 minutes at 4 °C in the dark. Based on this MoAb combination, MSCs were identified and sorted as SSC^hi^ CD13^high^ CD73^+^ CD105^+^ CD45^–^ CD34^–^ CD117^–^ cells with a purity systematically ≥95 %. In addition, those cells which did not meet the phenotypic criteria for CD13^high^ CD105^+^ CD45^–^ cells (i.e., non-MSCs) were also isolated; in this later fraction (non-MSC cell fraction) no contamination (<0.01 %) by cells phenotypically compatible with MSCs was detected. The sorted non-MSC and MSC fractions were separately resuspended in Dulbecco’s modified Eagle’s medium (DMEM; Sigma-Aldrich, Steinheim, Germany) supplemented with 20 % autologous plasma, L-glutamine 2 mM and 1 % penicillin-streptomycin (Gibco), and plated in six-well culture plates (Corning Inc., Corning, NY, USA). In parallel, isolation of MSCs using conventional MSC expansion after in vitro culture was also performed, as described elsewhere [1, 36]. Briefly, MNCs were resuspended in DMEM medium supplemented with 15 % FBS, L-glutamine 2 mM and 1 % penicillin-streptomycin, and plated in six-well culture plates at a concentration of 1 × 10^6^ cells/well. Culture plates were maintained in a humidified atmosphere with 5 % CO_2_ at 37 °C. After 4 days of cell culture (for both purified MSCs and MNCs), nonadherent cells were removed and fresh medium (DMEM plus 15 % FBS, L-glutamine 2 mM and 1 % penicillin-streptomycin) was added; for the sorted non-MSC fraction, cultured cells were washed and the culture medium replaced. These steps were then repeated every 2 days until the culture reached 80 % cell confluence. At this moment, nonadherent cells were discarded while adherent cells were detached with 0.05 % trypsin/EDTA (Gibco) and plated again in two or three culture flasks. This later procedure was repeated every 2 days, three times, in order to ensure that contaminating hematopoietic cells had been washed out of the cell culture.

### Adipogenic, chondrogenic and osteogenic in vitro differentiation of MSCs

In order to fulfill the ISCT criteria for the definition of MSCs [18], adipogenic, osteogenic and chondrogenic differentiation was performed on both sorted MSCs and MNC-derived MSCs, as previously described [17, 37]. Briefly, MSCs were resuspended and plated in a 24-well culture plate at a concentration of 1 × 10^4^ cells/well with either conditioned (differentiation) medium or with standard growth medium (DMEM plus 15 % FBS, L-glutamine 2 mM and 1 % penicillin-streptomycin); fresh medium was added every 2 days. To induce adipogenic differentiation, cells were incubated for 8 days with the STEMPRO® Adipogenesis Differentiation Kit medium (Invitrogen Life Science, Grand Island, NY, USA), according to the manufacturer’s instructions. The presence of neutral lipids was visualized by standard staining with Oil Red O (Sigma-Aldrich, St. Louis, MO, USA) [38]. In parallel, cells were also grown in osteogenic induction medium (STEMPRO® Osteogenesis Differentiation Kit medium (Invitrogen Life Sciences)) to induce osteogenic differentiation. Detection of osteoblasts was performed both after 8 days of culture using alkaline phosphatase staining [39], and after 11 days of culture through demonstration of the presence of calcium deposits using the alizarin red S (Sigma-Aldrich) staining [40]. To induce chondrogenesis, 2.5 × 10^4^ cells were placed in a 15-ml polypropylene tube and centrifuged (150 g for 10 minutes) in order to form a pelleted cellular micromass at the bottom of the tube. The cell pellet was cultured in 500 μl chondrogenic induction medium (STEMPRO® Chondrogenesis Differentiation Kit medium) following the recommendations of the manufacturer. Fresh chondrogenic differentiation medium was added every 2 days; after 27 days, the micromass was fixed, embedded in paraffin, cut in a microtome and stained with toluidine blue (Sigma-Aldrich) [41].

### Multiparameter flow cytometry immunophenotypic studies of in vitro expanded MSCs

For the assessment of the phenotype of in vitro expanded MSCs (i.e., the expression of those proteins previously evaluated on ex vivo MSCs) a direct immunofluorescence technique was used as previously described in detail [42]. Briefly, 2 × 10^5^ cells were stained (30 minutes at 4 °C in the dark) with eight-color combinations of MoAbs (Additional file 1: Table S1); stained cells were subsequently measured in a FACSCanto II flow cytometer equipped with the FACSDiva software.

### Statistical analyses

For all continuous variables, their median, mean, standard deviation, range and both the 25th and 75th, and the 10th and 90th percentiles, were calculated; for categorical variables, frequencies were reported. Statistical significance (*P* value ≤0.05) was determined by the non-parametric Kruskal-Wallis and Mann-Whitney U tests (for continuous variables in case of independent samples) or the Wilcoxon's and Friedman nonparametric tests for paired samples. For all statistical analyses the SPSS 18.0 software (SPSS, Chicago, IL, USA) was used.

## Results

### Frequency and ex vivo immunophenotypic features of CD13^high^ CD105^+^ CD45^–^ cells in normal and reactive BM samples

Overall, a CD13^high^ CD105^+^ CD45^–^ cell population was identified in every BM sample analyzed. Such cells were present at relatively low frequencies (median 0.028 %; range 0.0004–0.61 %) in all BM samples studied. Overall, they showed high but heterogeneous light scatter features, associated with lack of expression of early hematopoietic cell markers (CD34^–^, CD45^–^, CD117^–^), monocyte–macrophage related proteins (CD11b^–^, CD14^–^) and B cell lineage markers (CD19^–^) (Table 1 and Fig. 1). Conversely, expression of classical MSC-related markers (CD73, CD90, CD105 and CD146) was systematically observed in this cell compartment for all BM samples analyzed. Despite the above markers being detected in the whole CD13^high^ CD105^+^ CD45^–^ cell population (100 % of the cells; Table 1), different expression levels for individual antigens were observed for distinct markers; thus, CD146 was typically dimly expressed, whereas the pattern of expression of CD90 was heterogeneous among different samples (normalized MFI ranging from 1870 to 260,738 arbitrary fluorescence units), as well as among cells from individual samples (Fig. 1). Expression of CD105 and CD73 was also detected in 100 % of the cells with homogeneously positive-to-moderate positive expression levels (Fig. 1). Reactivity for CD10 and HLA-DR were heterogeneous and typically restricted to only a subset of the whole CD13^high^ CD105^+^ CD45^–^ cell population (62 % to 97 % and 50 % to 100 % of the CD13^high^ CD105^+^ CD45^–^ cells, respectively). Expression of other, more recently described, MSC-associated markers (nerve growth factor receptor (CD271), MSCA-1, SSEA-4 and STRO-1) was also assessed in a smaller subset of samples (n = 5). In these samples, co-expression of CD271, MSCA-1, SSEA-4 and STRO-1 was also systematically observed on CD13^high^ CD105^+^ CD45^–^ cells (Additional file 2: Figure S1). Nevertheless, whereas CD271 and MSCA-1 were strongly expressed on these cells, expression of STRO-1 was heterogeneous and SSEA-4 was only dimly expressed (Additional file 2: Figure S1).

**Table 1:** Ex vivo pattern of expression of individual markers on CD13^high^ CD105^+^ CD45^−^ cells from normal/reactive bone marrow samples

Code	Antigen	Expression pattern	% of positive cells^a^	Amount of protein expressed/cell^b^
		(% of positive cases; positive cases/total cases)		
CD10	Neprilysin	−/+	89 %	6916
		(100 %; 18/18)	(62–97 %)	(1883–22,177)
CD11b	Integrin alpha-M	–	NA	NA
		(0 %; 0/9)		
CD13	Aminopeptidase N	++/+++	100 %	122,414
		(100 %; 60/60)	(100–100 %)	(45,582–260,438)
CD14	Myeloid Cell-Specific Leucine-Rich Glycoprotein	–	NA	NA
		(0 %; 0/47)		
CD19	B-Lymphocyte Surface Antigen B4	–	NA	NA
		(0 %; 0/37)		
CD34	Hematopoietic Progenitor Cell Antigen-1	–	NA	NA
		(0 %; 0/60)		
CD45	Leukocyte common antigen	–	NA	NA
		(0 %; 0/60)		
CD73	Ecto-5′-nucleotidase	+/++	100 %	7816
		(100 %; 58/58)	(100–100 %)	(3268–23,090)
CD90	Thy-1	+/+++	100 %	21,875
		(100 %; 55/55)	(100–100 %)	(1870–260,738)
CD105	Endoglin	+	100 %	2463
		(100 %; 60/60)	(100–100 %)	(700–4620)
CD117	Kit	–	NA	NA
		(0 %; 0/54)		
CD146	Melanoma cell adhesion molecule	+Low	100 %	547
		(100 %; 55/55)	(100–100 %)	(267–3054)
NA	HLA-DR	−/+	91 %	3296
		(100 %; 34/34)	(50–100 %)	(390–15,823)

Codes used for the intensity of expression of individual markers: (−) negative; (−/+) heterogeneous from negative to positive; (+Low) dim positive (median fluorescence intensity (MFI) ≥250 and <1000); (+) moderate positive (MFI ≥1000 and <10,000); (++) positive (MFI ≥10,000 and <50,000); (+++) strong positive (MFI ≥50,000). Mixed symbols indicate variable reactivity, either among different samples or among cells from the same sample

^a^Results expressed as median (range)

^b^Normalized MFI values (median (range)) calculated after subtracting the background autofluorescence levels observed for each individual marker-associated fluorochrome and expressed as arbitrary units scaled from 0 to 262,000

*NA* not applicable

Of note, with the exception of the expression of CD90, which was significantly decreased in reactive (versus normal) samples, no differences were observed between normal and reactive BM MSCs for all other markers analyzed (Table 2).

**Table 2:** Distribution and phenotypic profile of CD13^high^ CD105^+^ CD45^–^ cells in normal versus reactive bone marrow samples

	Normal BM (n = 9)		Reactive BM (n = 51)	
Antigenic profile	% positive cells	MFI	% positive cells	MFI
% CD13^high^ CD105^+^ CD45^−^ cells	0.008 (0.0004–0.04)		0.03 (0.0007–0.6)*	
CD10	91 %	7624	88 %	6916
	(75–97 %)	(2069–22,177)	(62–97 %)	(1883–14,821)
CD11b	NA	NA	NA	NA
CD13	100 %	162,716	100 %	114,760
	(100–100 %)	(58,223–260,438)	(100–100 %)	(45,582–182,771)
CD14	NA	NA	NA	NA
CD19	NA	NA	NA	NA
CD34	NA	NA	NA	NA
CD45	NA	NA	NA	NA
CD73	100 %	7241	100 %	8149
	(100–100 %)	(3268–23,090)	(100–100 %)	(4462–16,288)
CD90	100 %	64,102	100 %	17,729*
	(100–100 %)	(12,081–260,738)	(100–100 %)	(1870–108,409)
CD105	100 %	2598	100 %	2463
	(100–100 %)	(700–3420)	(100–100 %)	(1299–4620)
CD117	NA	NA	NA	NA
CD146	100 %	733	100 %	502
	(100–100 %)	(292–2992)	(100–100 %)	(267–3054)
HLA-DR	98 %	4038	87 %	2589
	(78–100 %)	(390–11,726)	(50–100 %)	(407–15,823)

Results expressed as median (range) percentage of positive cells and MFI calculated after subtracting baseline autofluorescence levels

*P < 0.05 versus normal BM

*BM* bone marrow, *MFI* median fluorescence intensity (expressed as arbitrary fluorescence units scaled from 0 to 262,000), *NA* not appropriate (as these markers were absent on CD13^high^ CD105^+^ CD45^−^ cells)

### In vitro expansion, and osteogenic, adipogenic, and chondrogenic differentiation of CD13^high^ CD105^+^ CD45^–^ cells

**Fig. 2:**
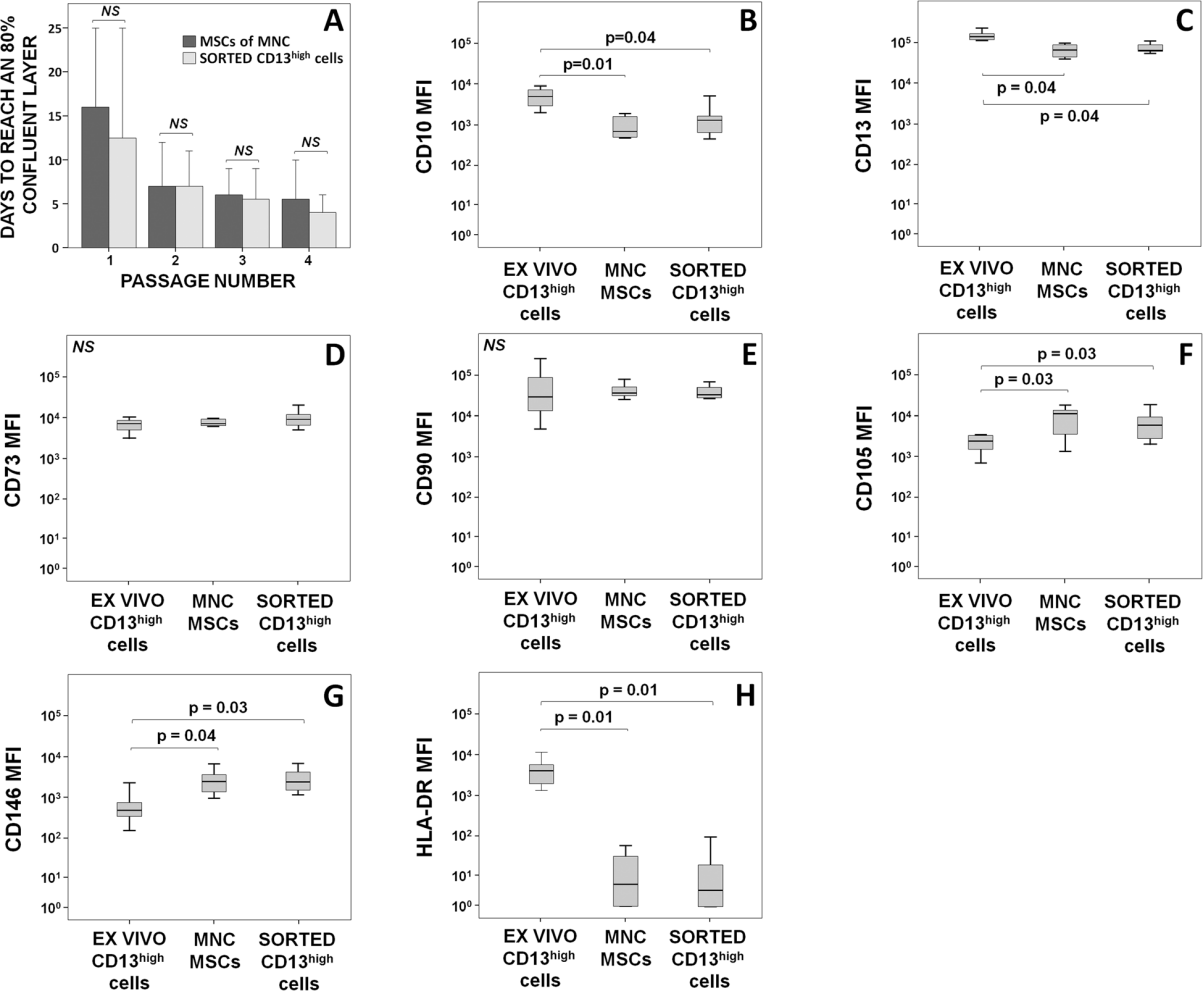
Growth kinetics (**a**) and immunophenotypic features (**b**–**h**) of mesenchymal stem cells (MSCs) obtained from BM mononuclear cells (MNCs) using a standard protocol for in vitro MSC expansion compared to the growth kinetics of FACS-sorted and in vitro cultured CD13^high^ CD105^+^ CD45^–^ BM cells (identified as CD13^high^ cells). **a** Bars and vertical lines correspond to median ± 95 % confidence interval values. **b**–**h** Notched boxes extend from the 25th to 75th percentile values; the lines in the middle and vertical lines correspond to median values and both the 10th and 90th percentiles, respectively. Normalized median fluorescence intensity (MFI) values are displayed after subtracting the background autofluorescence levels observed for each individual marker-associated fluorochrome channel (arbitrary units scaled from 0 to 262,000). *NS* not significant (*P* > 0.05)

Since the CD13^high^ CD105^+^ CD45^–^ BM cell population showed immunophenotypic features which were compatible with those of MSCs [18], we aimed to determine whether these cells also displayed the minimal functional criteria for MSCs (adherence to plastic and multipotent differentiation potential to the osteogenic, adipogenic and chodrogenic cell lineages), compared with donor-matched MSCs isolated from the same BM by the conventional method of density gradient purification followed by plastic adherence. In order to demonstrate that the CD13^high^ CD105^+^ CD45^–^ BM cell fraction contained virtually all BM MSCs, the negative BM cell fraction corresponding to all BM cells depleted on the CD13^high^ CD105^+^ CD45^–^ cell population was also cultured in parallel. Overall, both the FACS-sorted CD13^high^ CD105^+^ CD45^−^ cells and conventional in vitro cultured MSCs showed a similar ability to adhere to plastic; in addition, no significant differences were found regarding the growth rate of the two cells fractions (Fig. 2). In contrast, no adherent cells were observed in the BM cell fraction depleted from CD13^high^ CD105^+^ CD45^−^ cells at the time when the former two cell populations reached an 80 % confluent layer (passage 1).

**Fig. 3:**
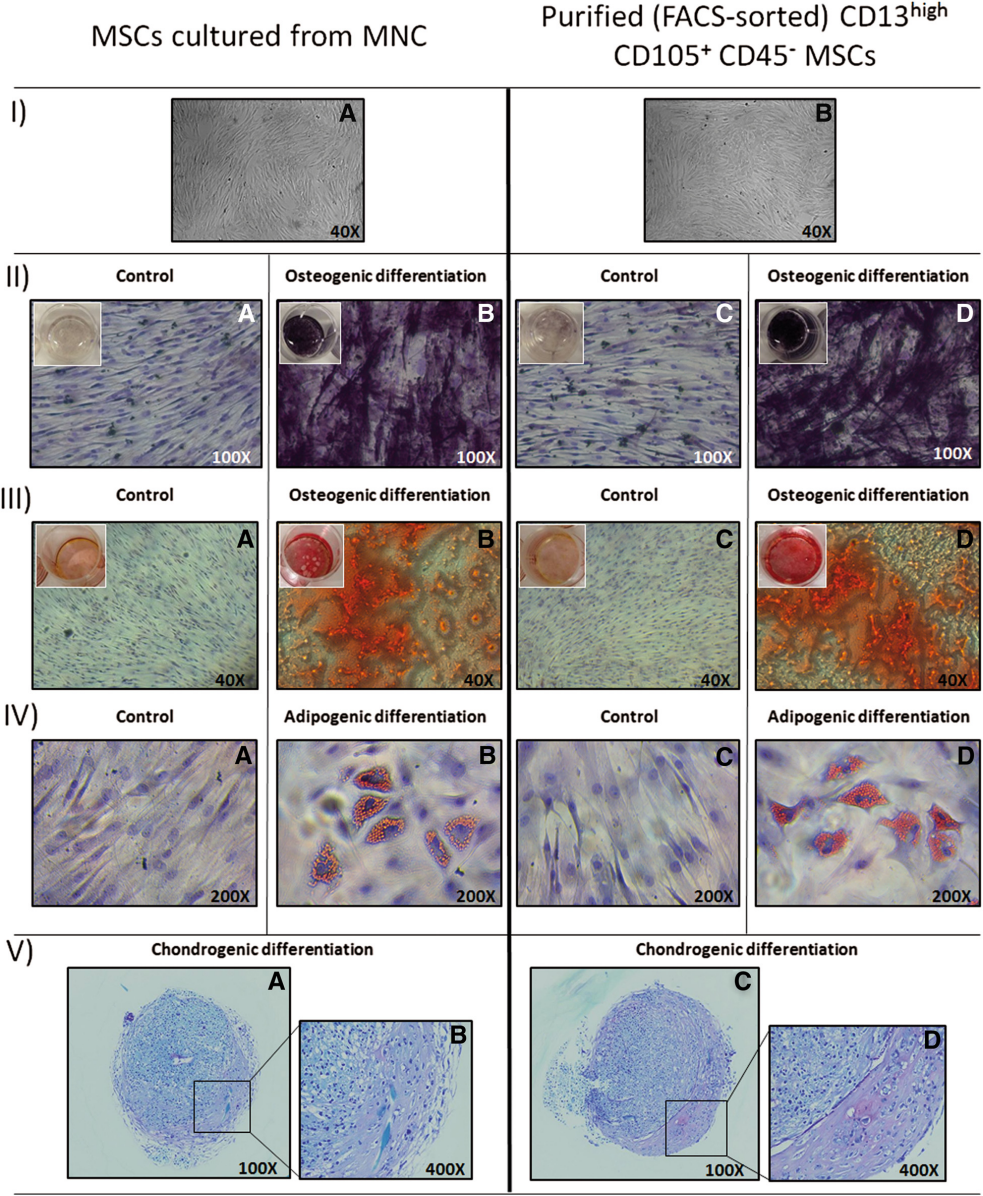
The functional features of mesenchymal stem cells (MSCs) observed in vitro after culture of Biocoll isolated mononuclear cells (MNCs) with conventional standard plastic adherence methods (*left panels*) versus FACS-purified and cultured CD13^high^ CD105^+^ CD45^–^ BM cells (*right panels*). Panels **I** *a* and **I** *b* show undifferentiated and unstained MSCs cultured for 24 days (passage 3). Panels **II** through **V** display cells which have been induced to differentiate in vitro towards the osteogenic (Panels **II** and **III**), adipogenic (Panels **IV**) and chondrogenic (Panels **V**) differentiation pathways. Osteogenic differentiation was assessed using alkaline phosphatase (Panel **II**) and alizarin red S (Panel **III**) stainings at days +8 and +11, respectively; adipogenesis was determined using the oil red O staining at day +8 (Panel **IV**), and chondrogenesis was evaluated by toluidine blue staining at day +27 (Panel **V**). For each staining technique in Panels **II**–**IV**, undifferentiated controls counterstained with hematoxylin are also displayed (Panels *a* and *c*)

The multipotent differentiation potential of both FACS-sorted CD13^high^ CD105^+^ CD45^–^ cells and MNC-cultured MSCs was evaluated in parallel in five different samples. Both fractions showed a normal osteogenic, adipogenic and chondrogenic differentiation after three passages, as assessed via: 1) determination of the alkaline phosphatase activity and the detection of extracellular calcium deposits using Alizarin Red staining (Fig. 3, panels II–III) after 8 and 11 days of culture with conditioned medium, respectively; 2) the Oil Red O staining for detection of lipid droplets after 8 days of culture with conditioned medium (Fig. 3, panel IV) and; 3) staining for cartilaginous extracellular matrix performed with toluidine blue after 27 days of culture with conditioned medium (Fig. 3, panel V).

### Immunophenotypic features of ex vivo and in vitro expanded FACS-sorted CD13^high^ CD105^+^ CD45^−^ cells versus MSCs obtained using conventional cell culture methods

Once compared to donor-matched MSCs isolated from BM MNCs by conventional plastic adherence methods, in vitro expanded FACS*-*sorted CD13^high^ CD105^+^ CD45^–^ cells showed similar immunophenotypic features. Thus, they displayed similar levels of CD10, CD13, CD73, CD90, CD105 and CD146, and at the same time both cultured cell populations lacked CD11b, CD14, CD19, CD34, CD45, CD117 and HLA-DR expression. In contrast, when FACS-purified and in vitro cultured CD13^high^ CD105^+^ CD45^–^ cells were compared with donor-matched (recently obtained, noncultured) ex vivo CD13^high^ CD105^+^ CD45^–^ BM cells, the former showed significantly higher levels of CD105 (*P* = 0.03) and CD146 (*P* ≤ 0.04) expression (Fig. 2f and g). In contrast, expression of both CD10 and CD13, despite remaining positive, was significantly decreased (*P* ≤ 0.04) after in vitro culture of CD13^high^ CD105^+^ CD45^–^ cells (Fig. 2b and c). Interestingly, expression of HLA-DR, which was detected on ex vivo BM CD13^high^ CD105^+^ CD45^–^ cells, was not detected on either of the two in vitro expanded donor-matched MSC populations (*P* = 0.01) (Fig. 2h).

## Discussion

In recent years, growing interest has emerged about MSCs due to their unique biological properties (e.g., their low immunogenicity, their immunomodulatory properties, availability, self-renewal and differentiation capacities) [1, 9, 43, 44] which make them a good candidate for cell therapy, as well as their involvement in the pathogenesis of several diseases [14, 15, 17]. However, the need for in vitro expansion of MNC fractions of BM cells for the definition of MSCs (which may affect the natural properties of these cells) together with the lack of (universally accepted) specific antigens and phenotypes for their ex vivo identification and positive isolation, has led to a growing interest in the identification of potential markers/phenotypes and the establishment of specific strategies for accurate in vivo and ex vivo identification of MSCs [28, 30]. In recent years, important advances have been achieved in this regard and several markers such as CD271 and MSCA-1 have been identified which can be used for purification of MSCs [32, 45–47]. However, some of these markers are dimly expressed and do not provide a sufficient definition of MSCs versus other cells in the sample (e.g., SSEA-4 or CD146) and/or their expression is not restricted to MSCs (e.g., STRO-1 is also expressed by nucleated erythroid cells) [32–34]. In addition, other markers have been found to be associated to, but not specific of, MSCs such as CD13, CD105 and CD90 [33, 34]. Here we show that the CD13^high^ CD105^+^ CD45^–^ immunophenotype defines a unique minor subset of BM cells that display MSC features which can be detected ex vivo on fresh BM aspirated samples prior to cell culture. Furthermore, we show that in vitro expansion of these cells modulates their immunophenotypic characteristics including the expression of markers currently used in the definition of MSCs; these results reinforce the need for a more detailed ex vivo characterization of MSCs.

In this study, CD13^high^ CD105^+^ CD45^–^ cells were systematically present in all normal/reactive BM samples analyzed. In the BM samples studied, these cells represented a minor cell compartment which displayed a unique and clearly different phenotype, distinct from that of all BM cell populations described so far. Therefore, these cells showed features distinct from those of B cells (CD19^–^), monocyte/macrophage lineage cells (CD11b^–^, CD14^–^), erythroid precursor cells (CD13^hi^, CD73^+/++^, CD90^+/+++^), hematopoietic precursor cells (CD34^–^, CD117^–^) and endothelial cells (CD34^–^), also lacking the expression of pan-leukocyte markers (CD45^–^) [18]. Further characterization of these cells revealed immunophenotypic features previously described to be associated with MSCs (e.g., CD10, CD73, CD90, CD146, CD271, MSCA-1, SSEA-4 and STRO-1 expression) [1, 18]. In fact, the immunophenotypic pattern of these CD13^high^ CD105^+^ CD45^–^ BM cells is fully compatible with the widely described MSC profile, except for the expression of HLA-DR which was found to be systematically expressed in all or a substantial fraction of all ex vivo studied CD13^high^ CD105^+^ CD45^–^ BM cells. Of note, HLA-DR has been previously reported to be absent on MSCs unless they had been stimulated (e.g., by interferon-γ) [18, 48]. CD13^high^ CD105^+^ CD45^–^ cells were isolated using a large set of markers, defined upon their ex vivo characterization in a large number of BM samples, in order to ensure a high cell purity. Based on this, sorted CD13^high^ CD105^+^ CD45^–^ BM cells showed similar growth kinetics, plastic adherence ability and osteogenic, adipogenic and chondrogenic differentiation potential as compared to those exhibited by donor-matched MSCs isolated using conventional in vitro culture methods. These results suggest that, in fact, this minor ex vivo BM CD13^high^ CD105^+^ CD45^–^ cell population corresponded to multipotent MSCs. In contrast, no adherent cells with MSC functions were observed in the BM cell fraction depleted on this specific minor cell population. These findings suggest that the CD13^high^ CD105^+^ CD45^–^ phenotype would identify the overall MSC population. Alternatively, MSCs could consist of a heterogeneous cell population which includes a pool of different cells with distinct proliferation and differentiation capacities [32], the MSCs not fulfilling the CD13^high^ CD105^+^ CD45^–^ criteria potentially displaying a lower proliferation and/or plastic adherence potential or being highly diluted and, consequently, requiring more time and/or distinct culture conditions to expand.

Of note, when immunophenotypic studies were repeated on expanded MSC fractions, no differences were observed between donor-matched in vitro cultured MSCs which had been isolated from the BM by the conventional density gradient (e.g. Biocoll) purification followed by plastic adherence method and in vitro expanded CD13^high^ CD105^+^ CD45^–^ (FACS-purified) cells. Interestingly, both in vitro expanded cell populations lacked expression of HLA-DR (previously detected on the CD13^high^ CD105^+^ CD45^–^ cells from all samples studied ex vivo), suggesting the ex vivo identified CD13^high^ CD105^+^ CD45^–^ cells in fact fulfill all minimal criteria for the definition of MSCs, since such immunophenotypic criteria were established on cultured cells [18]. In addition, since HLA-DR is known to be expressed on in vitro expanded MSCs upon activation [48], it could be argued that, for example, the BM extraction process could lead to activation of these cells, which could “return” to a resting state upon stabilization in culture. However, when both in vitro expanded fractions of MSCs were compared with their donor-matched ex vivo CD13^high^ CD105^+^ CD45^–^ cell population, for the remaining MSC markers, significantly higher expression levels were detected for CD105 and CD146 among the cultured cells, in association with lower CD10 and CD13 expression. These immunophenotypic differences may contribute to explain the lack of consensus regarding the antigenic profile (and also morphological characteristics) of BM MSCs in vivo, and at the same time they would support previous results suggesting in vitro modulation of the expression of several MSC-related markers (e.g., HLA-DR or CD146) after long-term cell culture [20, 21], particularly when different culture conditions are used for MSC expansion (e.g., CD10, CD73, CD90, CD105, CD146) [44, 49], or the processes associated with cell adherence and growth on plastic (e.g., CD44, CD34) varies [28, 50]. Such in vitro antigen expression-associated changes may potentially contribute to explain also some of the discrepancies reported in the literature as regards the genetic, phenotypic and functional properties of in vitro cultured MSCs [17, 22–27]. Consequently, the here described CD13^high^ CD105^+^ CD45^–^ MSC-specific phenotype may contribute to a more accurate and standardized ex vivo characterization of MSCs, and a better understanding of the in vivo biological and functional features of this cell population, therefore contributing also to establish more accurately the normal versus altered MSC immunophenotypic transcriptional and functional profiles in different disease conditions. Further studies, in larger series of BM samples from patients with distinct disease conditions, are necessary to fully characterize these cells and to determine whether the expression of CD13, CD105 and CD45 is stable enough to be used as a backbone combination for the identification and characterization of BM MSCs in patients with distinct hematological malignancies and other hematological and nonhematological disorders.

## Conclusions

In summary, here we demonstrate for the first time that BM MSCs can be identified ex vivo in normal/reactive BM, based on a robust CD13^high^ CD105^+^ and CD45^–^ inmunophenotypic profile. In addition, we also show that in vitro expansion of these cells is associated with significant changes in the immunophenotypic profile of MSCs. Further studies are needed for more detailed ex vivo characterization of these cells as a basis for the identification of altered genotypes and phenotypes in BM MSCs from patients diagnosed with different disease conditions, such as myeloid and lymphoid malignancies.

## Additional files

Additional file 1: Table S1. List of antibodies used for the identification, characterization and isolation of bone marrow mesenchymal stem cells, including CD13^high^ CD105^+^ and CD45^–^ cells. (DOC 41 kb) Additional file 2: Figure S1. Representative bivariate dot plots and single parameter histograms showing the ex vivo expression profile of widely-accepted markers (CD271, MSCA-1, SSEA-4 and STRO-1) on bone marrow CD13^high^ CD105^+^ CD45^–^ cells. Panels A through C show the gating strategy used for the identification of these bone marrow cells based on their unique CD13^high^ CD105^+^ CD45^–^ features. Panels D through G illustrate the immunophenotypic features of bone marrow CD13^high^ CD105^+^ CD45^–^ cells from a representative healthy donor. Panel H summarizes the expression of CD271, MSCA-1, SSEA-4 and STRO-1 on CD13^high^ CD105^+^ CD45^–^ cells from five healthy donors. Baseline autofluorescence and expression levels for each protein are indicated in the histogram plot in gray and black, respectively, using the overlay histogram function of the Infinicyt software. On panel H the open circles represent individual samples, short horizontal and vertical lines display median values and the 95 % confidence interval of the levels of antigen expression (MFI values), respectively. *SSC* sideward light scatter, *MFI* median fluorescence intensity (arbitrary units scaled from 0 to 262,000). (TIFF 1440 kb)

## Abbreviations

APC: Allophycocyanin
BM: Bone marrow
Cy5.5: Cyanin 5.5
Cy7: Cyanin 7
DMEM: Dulbecco’s modified Eagle’s medium
EDTA: Ethylenediaminetetraacetic acid
FBS: Fetal bovine serum
FITC: Fluorescein isothiocyanate
ISCT: International Society for Cellular Therapy
MFC: Multiparameter flow cytometry
MFI: Median fluorescence intensity
MNC: Mononuclear cell
MoAb: Monoclonal antibodies
MSC: Mesenchymal stem cell
PacO: Pacific orange
PBS: Phosphate-buffered saline
PE: Phycoerythrin
PerCP: Peridinin chlorophyll protein
