# Supplementary material for: Ex vivo identification and characterization of a population of CD13high CD105+ CD45− mesenchymal stem cells in human bone marrow
Source: Stem Cell Res Ther. 2015 Sep 7;6(1):169. doi: 10.1186/s13287-015-0152-8 (PMC4562124; doi:10.1186/s13287-015-0152-8)
Supplement: Additional file 1: Table S1. — List of antibodies used for the identification, characterization and isolation of bone marrow mesenchymal stem cells, including CD13high CD105+ and CD45– cells. (DOC 41 kb) [file 13287_2015_152_MOESM1_ESM.doc]

**Supplementary Table S1.** List of antibodies used for the identification, characterization and isolation of bone marrow mesenchymal stem cells, including CD13^high^ CD105^+^ and CD45^-^ cells.

| **Antibody conjugate** | **Clone** | **Source** |
| --- | --- | --- |
| **CD10- APC H7** | HI10a | BD Biosciences^1^ |
| **CD11b - FITC** | Bear1 | Beckman Coulter antibodies^2^ |
| **CD13 - APC** | WM15 | BD Biosciences^1^ |
| **CD14 – APC H7** | MφP9 | BD Biosciences^1^ |
| **CD19 - APC H7** | SJ25C1 | BD Biosciences^1^ |
| **CD34 - PerCP-Cy5.5** | 8G12 | BD Biosciences^1^ |
| **CD45 - PacO** | HI30 | Invitrogen^3^ |
| **CD45 - OC515** | GA90 | Cytognos^4^ |
| **CD73 - PE** | AD2 | BD Biosciences^1^ |
| **CD90 - PE** | 5E10 | BD Biosciences^1^ |
| **CD105 - FITC** | 166707 | R&D Systems^5^ |
| **CD105 - PE** | 1G2 | Beckman Coulter antibodies^2^ |
| **CD117 – PE Cy7** | 104D2D1 | Beckman Coulter antibodies^2^ |
| **CD146 - PE** | P1H12 | BD Biosciences^1^ |
| **CD271 – PE Cy7** | ME20.4 | Biolegend^6^ |
| **HLA-DR - PacB** | L243 | Biolegend^6^ |
| **HLA-DR - PECy7** | L243 | BD Biosciences^1^ |
| **MSCA-1- PE** | W8B2 | Miltenyi Biotec^7^ |
| **SSEA-4- PE** | MC-813-70 | Biolegend^6^ |
| **STRO-1- FITC** | STRO-1 | Biolegend^6^ |

APC, allophycocyanin; FITC, Fluorescein isothiocyanate; PerCP-Cy5.5, peridinin chlorophyll protein–cyanin 5.5; PacO, Pacific orange; OC515, Orange Cytognos 515; PE, phycoerythrin; PE Cy7, phycoerythrin cyanine 7; PacB, Pacific Blue.**^1^** BD Biosciences (BD, San José, CA, USA);^2^ Beckman Coulter (Brea, CA, USA);^3^ Invitrogen (Carlsbad, CA, USA);^4^ Cytognos SL, (Salamanca, Spain); ^5^  R&D Systems (Minneapolis, MN, USA);^6^ Biolegend (San Diego, CA, USA); ^7^ Miltenyi Biotec (Bergisch Gladbach, Germany)
